# Development of Chitosan/Rice Husk-Based Silica Composite Membranes for Biodiesel Purification

**DOI:** 10.3390/membranes12040435

**Published:** 2022-04-17

**Authors:** Saiful, Ulfa Riana, Muliadi Ramli, Muhammad Iqrammullah, Yanuardi Raharjo, Yusuf Wibisono

**Affiliations:** 1 Department of Chemistry, Faculty of Mathematics and Natural Science, Universitas Syiah Kuala, Darussalam, Banda Aceh 23111, Indonesia; ulfa.riana@mhs.unsyiah.ac.id (U.R.); muliadiramli@unsyiah.ac.id (M.R.); 2 Research Center for Environmental and Natural Resources, Universitas Syiah Kuala, Darussalam, Banda Aceh 23111, Indonesia; 3 Graduate School of Mathematics and Applied Sciences, Universitas Syiah Kuala, Banda Aceh 23111, Indonesia; m.iqhram@oia.unsyiah.ac.id; 4 Membrane Science and Technology Research Group, Chemistry Department, Faculty of Science and Technology, Universitas Airlangga, Surabaya 60115, Indonesia; yanuardiraharjo@fst.unair.ac.id; 5 Department of Bioprocess Engineering, Faculty of Agricultural Technology, Brawijaya University, Malang 65141, Indonesia; y_wibisono@ub.ac.id

**Keywords:** chitosan, crude biodiesel, inorganic–organic composite membrane, rice husk, silica

## Abstract

Inorganic–organic composite membranes (IOCMs) are an alternative separation method developed for their straightforward process, economic benefits, and ease of scaling up. The IOCMs in this study were prepared from a biopolymer chitosan matrix and rice husk-based silica filler to remove impurities from crude biodiesel. The IOCMs were prepared through phase inversions, in which the priorly prepared silica particles were dispersed in the dope solution of chitosan. The maximum loading of the silica particles was 60%, capable of reducing the soap level, free glycerol level, and acid number from 547.9 to 12.2 mg/L, 54 to 0.041%, and 2.02 to 1.12 mgKOH/g. These reduced impurity values have satisfied the standardized quality. The chemical composition and morphology of the IOCM was characterized using Fourier-transform infrared spectroscopy and scanning electron microscope–energy dispersive X-Ray spectroscopy. The IOCM water absorption-based porosity and swelling degree were studied as well. Further investigation using isothermal modeling revealed the adsorption dependency against the Sips model equation (R^2^ = 0.99 and root-mean-square errors = 1.77 × 10^−8^). Even though regeneration is still a challenging factor in this study, the IOCM prepared from chitosan and rice husk-derived silica particles could be used in crude biodiesel purification.

## 1. Introduction

Biodiesel is a product of methyl esters produced from transesterification reactions of fatty acids or plant oils [1]. Biodiesel produced from the transesterification reaction still contains these impurities and is not qualified according to the Indonesian National Standard (SNI) or international standard ASTM D6751 (North America), EN14214 (Europe) [2,3]. The small content of impurities in biodiesel might cause problems during its use in internal combustion engines and storage [4,5]. The problems include engine oil degradation, water-induced corrosion, blockage of fuel injectors due to soap, and engine leakage due to alcohol. Moreover, the impurity could induce smoke containing acrolein, a hazardous photochemical compound. Therefore, before being used as fuel, biodiesel must be purified from free fatty acids, glycerol, soap, and the catalyst leftover [6,7].

A promising method for biodiesel purification is the utilization of inorganic–organic composite membranes (IOCMs) for the filtration and adsorption processes [8]. Compared to the conventional ultrafiltration membrane for biodiesel purification, the novel membrane application in this study is also known as a membrane adsorber because it is embedded with adsorbent particles [3,9]. Recently, in renewable fuels, IOCMs have been developed using different membrane matrices to remove water content such as polyethersulfone [10] and polydimethylsiloxane [11]. Developed IOCMs have achieved a high removal of more than 90% of impurities, especially glycerol, during biodiesel production [12]. In comparison to the conventional wet washing method, biodiesel purification using an IOCM could practically avoid the excessive use of water and wastewater yield [1]. Nonetheless, the previous reports often used commercial materials to construct the IOCM, resulting in expensive and non-renewable materials.

Bio-based adsorbents, after the activation process, could be used for the removal of water and organic and inorganic compounds; therefore, they are compatible with biodiesel purification. A potential biomass-derived adsorbent is silica, which has been reported to work on a wide spectrum of impurities [13]. Silica could be obtained either from the synthesis process or through isolation from biomass such as rice husks. Silica derived from rice husks could be obtained straightforwardly through the process of ignition and extraction using alkalis. Silica itself has been famously applied in the purification of biodiesel [14], attributed to its large surface area and inert properties [15]. Using the agricultural biomass, rice husk, as a feed material for silica allows for an inexpensive production cost and supports the circular economy.

To increase its practical aspect, the silica can be embedded into the chitosan membrane matrix [8]. Chitosan can be obtained from chitin through a deacetylation reaction, resulting in a multifunctional polymer with N- and O-containing functional groups. These functional groups are responsible for the excellent performance of an adsorbent in the separation process [16,17,18]. In multiple reports, chitosan has shown its ability to remove heavy metals [17,19], biodiesel impurities [3], dyes [20], and pesticides [21]. Furthermore, chitosan can easily dissolve in acetic acid, allowing a simple and eco-friendly process for its membrane matrix preparation [22].

In this study, chitosan–silica-based IOCMs for biodiesel purification were developed, indicated by the reduction of free glycerol and acid number. The combination of silica and chitosan for biodiesel purification purposes has been scarcely reported. Granulated commercially available chitosan–silica biosorbent has been investigated for its application in biodiesel impurities removal [23]. The impurities contained in biodiesel upon its production include salts, soaps, methanol, and residual fatty acids (or glycerol). Silica, however, could be derived from rice husk waste. A study conducted in Indonesia suggested that rice husk ash could yield silica of around 60% (*w*/*w* rice husk ash) through an extraction using alkaline solvent [24]. The applications of silica deriving from rice husk have been reported multiple times for the adsorptive entrapment of various compounds [25,26,27]. However, in terms of biodiesel production, rice husk-derived silica has only been commonly applied as the catalyst—not for the adsorption of biodiesel impurities [28,29]. In the present study, sodium hydroxide was used to extract the silica from rice husk ash, which has been reported to produce a silica adsorbent with a high microporosity and surface area [30]. Furthermore, the silica was embedded in a polymeric membrane, allowing easier separation after the batch purification and filtration process.

## 2. Materials and Methods 

### 2.1. Materials

Chitosan was purchased from Fluka (Tokyo, Japan), with an average degree of acetylation of up to 94 mol%. Acetic acid 1% was used as a solvent and dimethyl formamide was used as an additive. HCl and NaOH were used as a washing solution and extraction solution of silica particles, respectively. KOH, ethanol, and pp indicators were used for acid number analysis. Acetone and indicators of blue bromophenol were used for soap level analysis. Chloroform, periodic acid, sodium thiosulfate, potassium iodide, and distilled water were used in free glycerol analysis. Unless otherwise stated, all chemicals used were pro-analysis grade and purchased from Merck (Jakarta, Indonesia). Rice husk waste (silica feed) was obtained from the rice mill in Aceh Besar, Indonesia. The crude biodiesel in this study was prepared from used cooking oil (Banda Aceh, Indonesia).

### 2.2. Silica Preparation from Rice Husk

**Figure 1:**
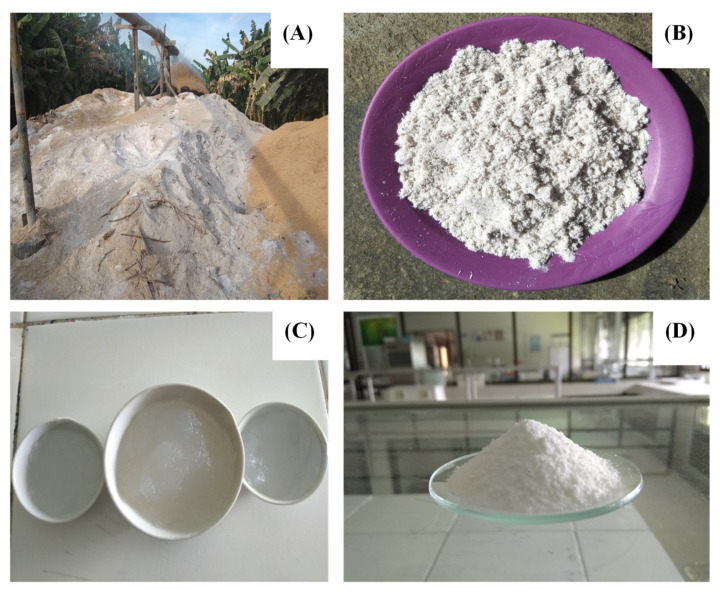
Rice husk ash can be processed into silica particles. (**A**) Bulky rice husk ash, (**B**) rice husk ash sample, (**C**) silica gel obtained from rice husk ash, (**D**) silica particle prepared from rice husk.

The silica extraction was carried out according to a previous procedure [31]. Briefly, the rice husk ash was washed with distilled water and then soaked in HCl 1 N solution for ± 24 h in a closed container to remove impurities. Soaked rice husk ash was then filtered and washed with boiling water to neutralize the pH. Thereafter, the rice husk was furnaced at 700 °C for 4 h to vaporize the organic contents. The rice husk ash was then refluxed using NaOH 1 N at 110 °C for 4 h to extract the silica. This step yielded a filtrate that was left to cool to room temperature and neutralized with HCl 1 N solution. The formed gel was filtered and oven-dried at 110 °C until the solid white silica particles were produced. The procedure steps of rice husk-derived silica particles have been presented in Figure 1.

The crystallinity analysis using a Shimadzu X-ray diffractometer (XRD)-700 (Kyoto, Japan) and functional group analysis using a Shimadzu 8400 Fourier transform infrared (FTIR) spectroscope (Kyoto, Japan) were carried out on the prepared sample. Micro-surface images of the silica particles were produced by scanning electron microscopy–electron dispersive spectroscopy (SEM-EDS) using a JEOL JSM 6510 LA (Tokyo, Japan). Furthermore, the surface area of the prepared silica was analyzed using Brunauer–Emmett–Teller (BET) and Barrett–Joyner–Halenda (BJH) isotherm equations based on the nitrogen adsorption–desorption at 77.3 K on QuadraSorb Station 1 (ver. 5.06).

### 2.3. Preparation of Inorganic–Organic Composite Membrane 

Chitosan with a concentration of 3% (*w*/*v*) was added to a mixture of dimethylformamide 10% (*v*/*v*) and acetic acid 1% (*w*/*v*). The mixture was stirred in a tightly sealed Erlenmeyer for ±24 h at room temperature and 250 rpm. Silica particles (varied from 10–60% *w*/*w*) were added to the previous mixture to obtain the IOCM. The doping solution was then printed on a glass plate. The solvent was allowed to evaporate at room temperature for 24 h. The membrane obtained was washed with NaOH 1% (*w*/*v*) and rinsed with distilled water, then air-dried at room temperature overnight. The membrane was cut to 2.5 cm^2^ for further use.

For the membrane samples, either IOCM or neat chitosan, the instrumental characterizations were carried out using the Shimadzu 8400 FTIR spectroscope, JEOL JSM 6510 LA for SEM-EDS (Tokyo, Japan), and Shimadzu XRD 7000 (Kyoto, Japan). Since the samples were in a film shape, the sample preparation prior to the analysis followed our previously published works [18,32].

### 2.4. Determination of Porosity and Swelling Degree 

The porosity and swelling degree determinations were based on the absorption of distilled water into the prepared membranes. The porosity (ε) and swelling degree (sd) values were calculated using Equations (1) and (2), respectively.

(1) $$\varepsilon \left(\%\right)=\frac{V_{\;wet}-V_{dry}}{V_{wet}}\times 100$$

(2) $$sd\left(\%\right)=\frac{V_{wet}-V_{dry}}{V_{dry}}\times 100$$

where *V_dry_* is the volume of the dry membrane and *V_wet_* is the wet volume membrane after being immersed in a water bath for 24 h at room temperature.

### 2.5. Membrane Pure Water Flux

The clean water flux experiment was determined using a dead-end Amicon type system filtration cell. The experiment was determined at room temperature. The device was pressured at 1 bar so that the feed solution flowed through the membrane. The reported flux values were measured at steady-state conditions and stirred under 200 rpm. The average flux value was calculated using several membrane pieces. The membrane water flux was calculated using the following equation:

(3) $$\mathrm{Flux}\;\left(J\right)=\frac{\mathrm{Volume}\;\mathrm{permeate}}{A\;\times \;t}$$

where J is the water flux (L/m^2^ h), V is the volume of the permeate (L), t is the time (h), and A is the membrane surface area (m^2^).

### 2.6. Batch Adsorptive Purification

The performance of the prepared IOCM was evaluated toward biodiesel purification employing a batch system. Three sheets of the membrane (2.5 cm^2^) were weighed and put in an Erlenmeyer containing 25 mL crude biodiesel. The purification was carried out at 200 rpm using a rotary shaker at room temperature. The contact times of 10, 20, 30, 60, and 120 min were used to obtain the optimum contact time carried out using IOCM with a silica particle load of 10%. Afterward, the study was continued with the investigation on the effect of particle loading employing the optimum contact time obtained previously. The adsorption isotherm studies were carried out under the optimum contact time and particle loading with glycerol concentrations of 0.01, 0.05, 0.1, 0.15, 0.2, 0.25, and 0.3%. Acid number, soap level, and free glycerol content were calculated before and after the batch purification treatment.

### 2.7. Biodiesel Filtration

The filtration performance of the flat sheet IOCM was measured at a constant permeation rate using a pressurize stirred dead-end filtration cell. The experiment was carried out using a membrane area of 19.62 m^2^ at a pressure of 1.5 bar. The permeate was collected and the free glycerol content, acid number, and soap concentration in the permeate samples were analyzed by a standard method.

### 2.8. Determination of Acid Number, Soap Levels, and Free Glycerol Content

#### 2.8.1. Acid Number

As much as 5 g of biodiesel was added to 10 mL of ethanol, followed by the addition of 3 drops of phenolphthalein. The mixture was titrated using 0.1 M KOH until a permanent pink color was formed. Acid numbers were calculated using the following Equation (4):

(4) $$\mathrm{Acid}\;\mathrm{number}=\frac{56.1\times N_{KOH}\times V_{KOH}}{m}$$

where $V_{KOH}$ is the volume of KOH solution used (mL), $N_{KOH}$ is the normality of the KOH solution in titration, and m is the mass of the biodiesel (g).

#### 2.8.2. Soap Levels

Five grams of biodiesel was added into 5 mL of acetone, followed by 3 drops of bromophenol blue (0.4% in water). Then, the mixture was titrated with 0.01 N HCl until the color turned yellow. The soap level (as sodium oleate) was calculated with Equation (5).

(5) $$\mathrm{Soap}\;\mathrm{number}=\frac{304,000\times N_{HCl}\times V_{HCl}}{m}$$

where $V_{HCl}$ is the volume of the HCl solution (mL) used in titration and $N_{HCl}\;$ is the normality of the HCl solution.

#### 2.8.3. Free Glycerol Content

Ten grams of biodiesel was added into a mixture containing 100 mL of chloroform and 50 mL of distilled water. The mixture was vigorously shaken for 30–60 s and left until the organic layer (chloroform) and the water layer were entirely separated. The procedure was followed by the addition of 3 mL of distilled water and 2 mL of periodic acid, then was left for 30 min. Then, 0.5 mL of KI was added and titrated with 0.01 N sodium thiosulfate until the brown color faded. Thereafter, the mixture was added with starch until the color turned blue. Then, it was titrated again until the color changed. The determination of total glycerol levels was calculated based on Equation (6).

(6) $$\mathrm{Gtb}=\frac{2.302\left(B-C\right)N_{sodium\;sulphate}}{m}$$

where Gtb is the free glycerol content in the sample, *B* is the volume of sodium sulfate for the blank, *C* is the sodium sulfate volume of the samples, and $N_{sodium\;sulphate}$ is the normality of the sodium sulfate.

### 2.9. Regeneration

The membrane was assumed to contain biodiesel impurities after being removed from the batch purification process. The membranes with biodiesel impurity contents was recovered using an organic solvent, methanol, as suggested previously [3]. The membrane was immersed into 250 mL of methanol in a sealed container at room temperature for 24 h. Afterward, the membrane was removed and rinsed with ultrapure water to wash out the remaining methanol until a neutral pH was achieved. The regenerated membrane was tested for the subsequent cycles of batch purification using the optimum conditions obtained earlier.

## 3. Results and Discussions

### 3.1. Isolation of Silica from Rice Husks 

**Figure 2:**
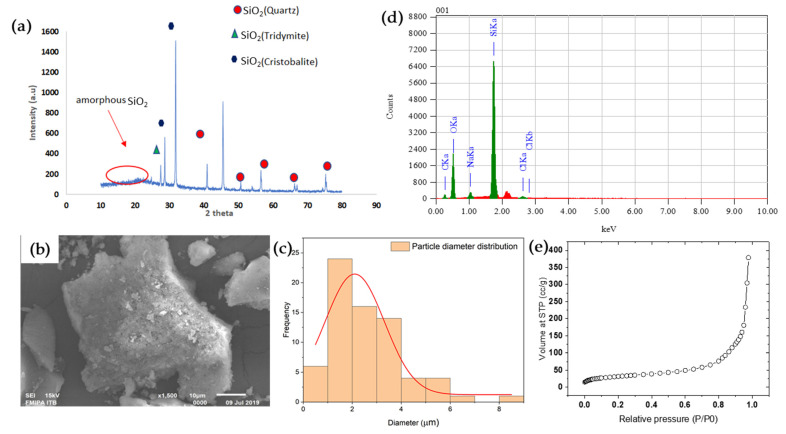
XRD pattern (**a**), SEM image (**b**), particle size distribution (**c**), EDS spectra of rice husk-derived silica particles (**d**), and BET isotherm curve of N_2_ adsorption onto the rice husk-derived silica particles (**e**). STP, standard temperature and pressure.

Figure 2a shows the X-ray diffractogram of the extracted silica from rice husk. The analysis shows dominant peaks of 2θ at 21–22°, indicating the success of the silica particle extraction with a high purity (>94%), as suggested by other studies [26,28]. The broad peak indicates the semi-crystalline properties of the material; silica in the form of amorphous, quart, critobalite, and tridymite. 

The SEM images (Figure 2b) show the particles have a rough surface covered with a flake-like structure. The particle shape is observed to be rather irregular, suggesting its ability to form a rough surface on the chitosan matrix during the preparation of the silica-embedded chitosan IOCM. Using ImageJ software on the SEM images, the determination of the particle diameter revealed the average particle size of 2.1 μm (Figure 2c). The silica particles prepared in this study are suitable for embedment onto the chitosan matrix with such a particle size. Furthermore, the EDS analysis (Figure 2d) substantiated the dominant percentage of Si and O content (the sum content of Si and O occupying > 94% of the total weight). The Si and O content could appear in the form of Si–O, Si–O–Si, or Si–OH functional groups, which contribute to the adsorbent active sites [33]. The next section will further discuss the analysis of these functional groups.

Based on the N_2_ adsorption using BET analysis, the surface area of the prepared silica particles was 113 m^2^/g (Figure 2e). Meanwhile, the pore diameter and pore volumes were 21 nm and 0.58 mL/g, respectively, according to BJH-modelled N_2_ desorption. The surface area obtained in the rice husk-based silica was comparatively higher than that in wheat husk-based silica, which only reached 0.67 to 0.92 m^2^/g [34,35]. These data suggest the sufficiency of the silica adsorbent to remove the impurities from the transesterification product. However, the prepared silica fillers’ BET surface area was lower than that of previously reported carbonaceous adsorbents [36,37]. Reported studies have agreed that the surface area of an adsorbent plays a significant role in the purification process. This further leads the focus of adsorbent activation to enhance the surface area [36]. Overall, the characteristics of the silica prepared from rice husk suggest the excellent adsorbent potential.

### 3.2. Thickness, Porosity, and Swelling Degree of the Prepared Membrane

The IOCM membrane in this study was prepared by adding the silica particle filler obtained and characterized previously. The IOCM was found to be thicker than the neat chitosan membrane. The neat chitosan membrane had a thickness of 0.045 mm, which was much lower than the IOCM with a thickness of 0.110 mm. This difference in thickness is due to the fact that the silica particle added in the chitosan matrix had a higher volume (lower density). The silica particles are a porous material which consequently possess a lower density [30]. Therefore, even though the weight of the doping solution of each membrane was casted on the same sized mold, the thickness of the IOCM increased. However, the IOCM chitosan–silica porosity and swelling degree were lower than that of the neat chitosan membrane. The IOCM had a 14.4% porosity and 18.6% swelling degree. These values were lower than the porosity and swelling degree of the pure chitosan membrane, which were 40.8% and 53.9%, respectively. The higher porosity and swelling degree of the neat chitosan is due to its hydrophilicity, allowing more water absorption [38]. In terms of filler embedment, a lower swelling degree is expected to prevent fillers from leaching out from the membrane [39]. Since the silica particle plays a significant role in adsorption, as opposed to chitosan, a lower swelling degree is preferable. Further characteristics using instrumental analysis such as SEM-EDS and FTIR are discussed in the following.

### 3.3. Membrane Characteristics

**Figure 3:**
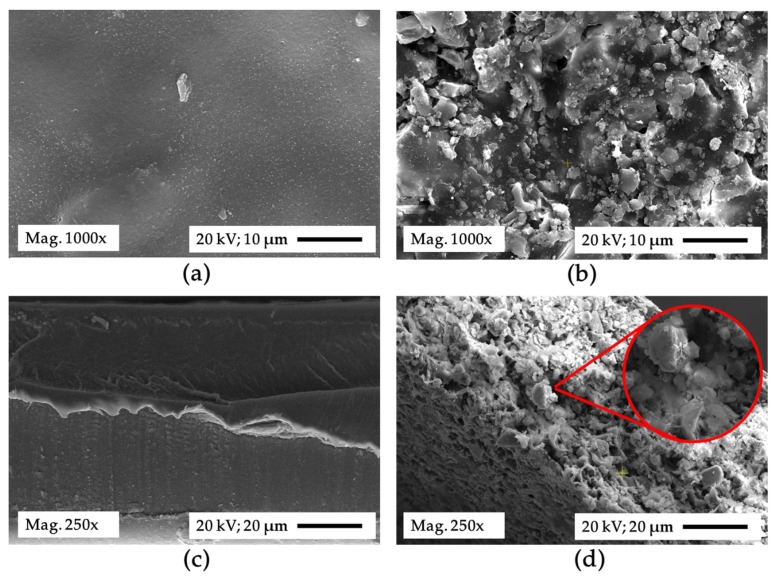
Surface images of chitosan (**a**) and the prepared IOCM (60% silica) (**b**). Cross-section images of chitosan (**c**) and IOCM (60% silica) (**d**). Surface and cross-section images were observed under SEM with 1000× and 250× magnifications. Highlight in red in the cross-section image of IOCM shows its appearance in 750× magnification.

The embedment of silica particles synthesized from rice husk contributes to the adsorptive feature of the IOCM. By modifying the membrane surface, the membrane morphology before and after the filler addition was captured using SEM under 1000× and 250× magnifications for the surface and cross-section parts, respectively (Figure 3). A smooth and dense surface could be observed in the chitosan membrane surface, which the cross-section image further corroborates. Following the filler addition, the membrane surface becomes rich in embedded particles of irregular shapes affected by the shape of silica particles or their agglomerates. The presence of a particle in the chitosan matrix leads to the formation of a rougher surface, which has been known to facilitate impurities uptake [38,40]. In a study employing bio-charcoal as a filler, a similar finding was reported, where the membrane appeared on a rougher surface and was attributed to higher adsorption than neat chitosan [40]. Moreover, the cross-section SEM image also shows the bulk structure of the IOCM governed by tiny irregular pores surrounded by silica particles. In general, the bulk porosity of the IOCM tends to be less than that of neat chitosan membranes since filler addition contributes to a higher membrane density [41,42]. 

**Figure 4:**
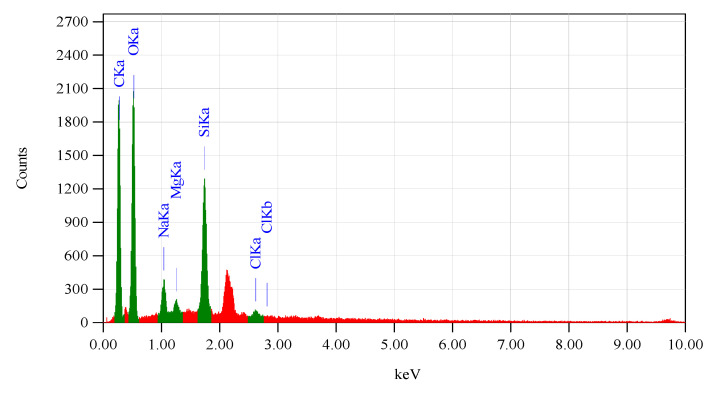
EDS data of the IOCMs containing silica particles from rice husk.

Figure 4 shows the elemental composition of the IOCMs from EDS data. Carbon (37.45%) and oxygen (49.42%) were two major elements in the IOCM, the main components of the organic chitosan membrane. The silica then follows with a percentage reaching 9.33%, contributed from the silica particle. Other elements including Na (2.29%), Mg (0.79%), and Cl (0.71) were also detected. This analysis demonstrates that the filler remained and immobilized in the membrane matrix during the fabrication. These findings are similar to those of previous reports, where silica particles were successfully embedded onto the matrix membrane [3].

**Figure 5:**
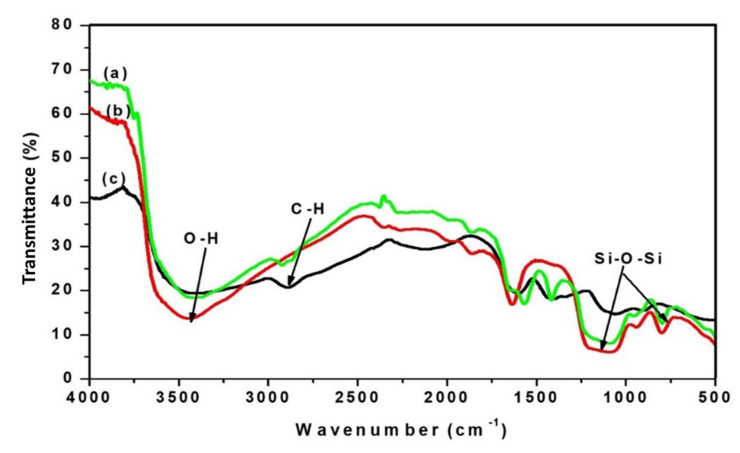
FTIR spectra of the chitosan–silica IOCM (**a**), silica particles (**b**), and neat chitosan (**c**).

Other IOCM characteristics are shown in the FTIR spectra (Figure 5) compared to the those of the silica particles and pure chitosan membrane. It can be seen that the spectrum of the IOCM (a) is a composite spectrum between the spectrum of the silica particles (b) and pure chitosan membrane (c). The functional groups of IOCMs were identified by absorption at a wavenumber of 957 cm^−1^ for Si–O stretching vibration and a wavenumber of 1092 for Si–O–Si symmetrical stretching vibration, as well as the –OH silanol group at a wavenumber of 3422 cm^−1^. The chitosan content is attributed to the absorbance at 1634 cm^−1^ (–C=O carbonyl group), 1570 cm^−1^ (bending vibrations of NH groups), 1419 cm^−1^ (bending vibration of CH group), and 1924 cm^−1^ (stretching vibration of aliphatic -CH group). The spectral peak at around 1600 cm^−1^ from the silica sample, assigned for the C=O carbonyl group, probably originates from the leftover of the charring process of the rice husk (corroborated by a small peak of C in the EDS spectra—Figure 2). The wavenumbers observed herein resemble that of a reported study developing a silica-based aldehyde chitosan hybrid material [23].

### 3.4. Study of Prepared Membrane on Crude Biodiesel Purification

#### 3.4.1. Effect of Contact Time 

**Figure 6:**
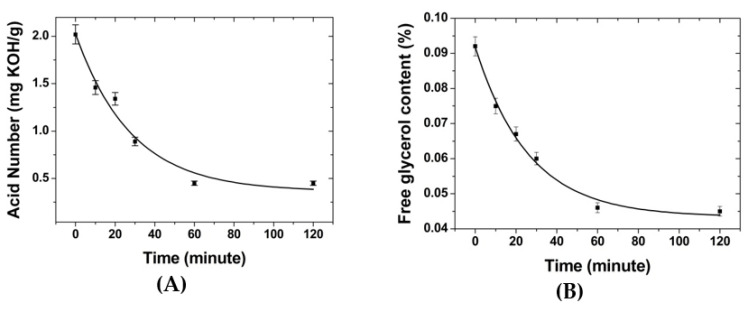
Effect of contact time on the reduction of (**A**) free glycerol content and (**B**) acid number in biodiesel.

The acid number and glycerol levels in biodiesel significantly decreased during the first 60 min (Figure 6), associated with the high diffusion force due to the unoccupied binding site. At this stage, the adsorption was dominated by the physical forces rather than chemical interaction [16,32]. The decrease in free glycerol content and the acid number was much less significant and tended to become saturated after reaching the 120th minute. This indicates that at the 60th minute, the binding site of the adsorbent had been fully occupied, and equilibrium of adsorption–desorption occurred. This finding is similar to the previous study using an IOCM for biodiesel purification that obtained the optimum contact time during the first hour of batch adsorption [3,9]. Therefore, the batch adsorption procedure was set for 60 min for further investigations.

#### 3.4.2. Effect of Silica Particle Loading

**Figure 7:**
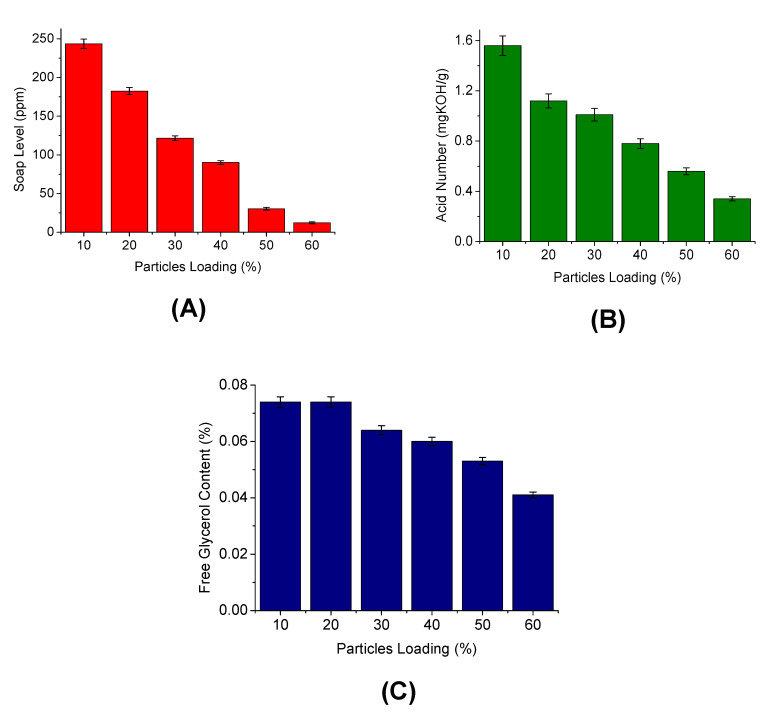
Effect of differences in loading silica particles on the quality of biodiesel: (**A**) soap level, (**B**) acid number, and (**C**) free glycerol content.

The effect of silica particle loading on the IOCM performance in the depletion of soap content, free glycerol level, and the acid number is presented in Figure 7. The impurities uptake was higher when more silica particles were loaded onto the chitosan membrane matrix. The IOCM containing 60% *w*/*w* silica particle decreased the soap level, free glycerol level, and acid number from 547.9 to 12.2 mg/L, 54 to 0.041%, and 2.02 to 1.12 mgKOH/g, respectively. The criteria of the final product meet the Indonesian National Standards (SNI), where the soap level is <50 ppm and the acid number is <0.5 mgKOH/g. The results of the biodiesel purification were similar to those reported by a study using commercial silica embedded on granular chitosan [23]. 

This study revealed that the biodiesel impurities adsorptive removal depends on the silica particle content. The adsorption of impurities onto silica may be associated with several interactions, such as chemical bonding, hydrogen bonding, hydrophobic bonding, or van der Waals. The hydrogen bond is the common interaction mechanism in this case, in which silica oxide (SiO_2_) may attract the impurity molecules with O-containing moieties via hydrogen bonding [33]. SiO_2_ may also undergo reconstruction and react with the water molecule, forming a silanol group (SiOH). This group can form a complex with water molecules (SiOH–OH_2_–OH_2_) [43]. The chitosan matrix may form interactions with the free acid, glycerol, and water molecules that, along with the silica particles, synergistically purify the biodiesel [3,23]. In our previous study using the rice husk-derived silica particles alone to purify the biodiesel (with the same weight of adsorbent), the decrease of acid number only reached around 30% [31]. Comparatively, in the present study, the acid number decreased as much as 44.6%, suggesting the synergistic action between the silica filler and chitosan matrix. 

#### 3.4.3. Adsorption Isotherm

**Figure 8:**
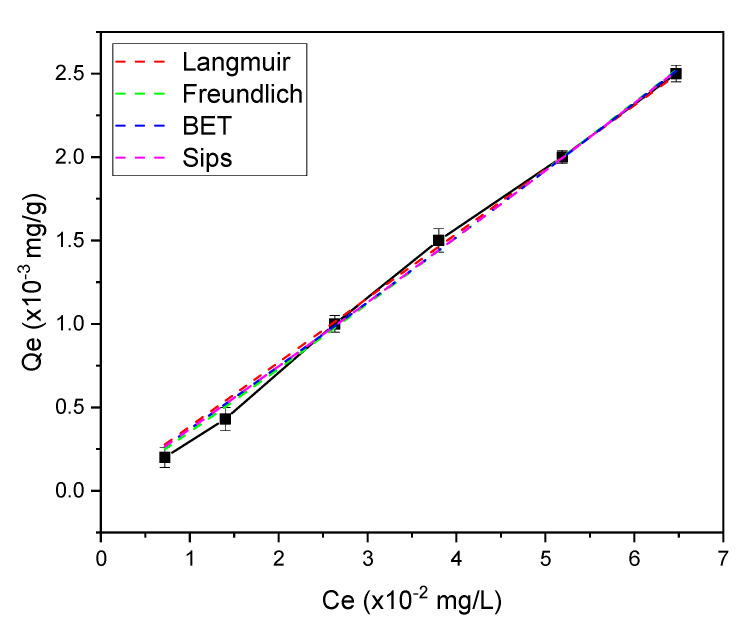
Non-linear fitting of Langmuir, Freundlich, BET, and Sips adsorption isotherm models. The solid line represents the experimental data and dashed lines represent theoretical data.

The adsorbate–adsorbent interaction during the impurities removal from crude biodiesel can be predicted using various isotherm models. Herein, we used Langmuir, Freundlich, BET, and Sips isotherm models based on the plot of adsorption capacity at equilibrium (Qe) vs. concentration at equilibrium (Ce), obtained from glycerol adsorption (Figure 8). Both studies using Langmuir and Freundlich isotherm models generate a good curve fitting with the coefficient of determination (R^2^) of 0.996 and 0.997, respectively. In this case, the assumption from both isotherm models may be applied to the experimental data [18,40]. Hence, we further studied the adsorption isotherms using three parameters—BET and Sips models that combine Langmuir and Freundlich models. The data of adsorption isotherm studies are presented in Table 1. We then found that the Sips model is the best isotherm model to predict the impurities adsorption onto the IOCM with R^2^ = 0.999 and root-mean-square errors (RMSE) = 1.77 × 10^−8^. This isotherm model is suitable for predicting adsorption using adsorbent with a heterogenous surface, which has a better accuracy at increased concentrations than the Freundlich model [44]. The Sips isotherm model exponent ($\beta_{S}$) of 1.00048 explains the linearity of the Qe vs. Ce curve.

### 3.5. Regeneration of the Prepared IOCM

**Figure 9:**
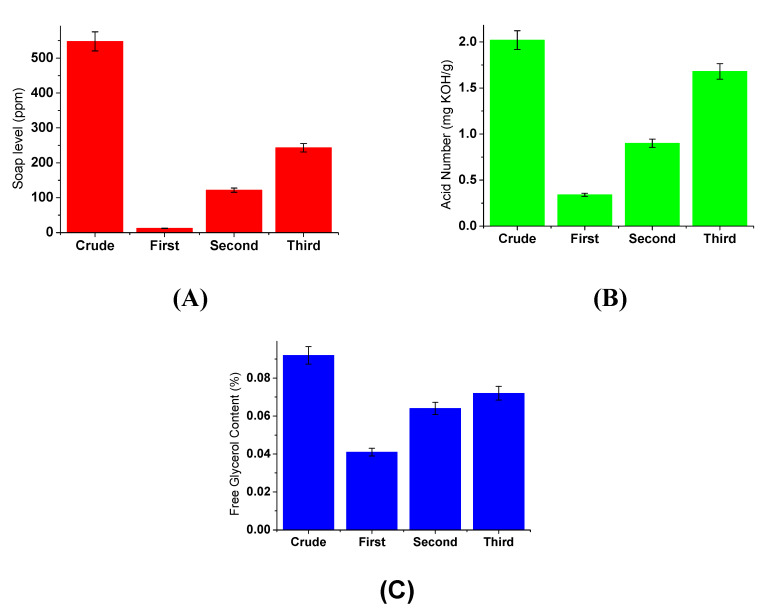
The adsorption capacity of regeneration of the IOCM of chitosan-silica: (**A**) soap level, (**B**) acid number, and (**C**) free glycerol content.

Membrane regeneration was carried out by soaking the used membrane with methanol for 24 h. In a previous study [3], it has been reported that the use of methanol is better than ethanol to regenerate membranes in biodiesel purification. Methanol has a smaller molecule size and more polar solvent so that it easily attracts impurities found in the IOCM of chitosan–silica. The results of biodiesel quality testing by adding the first and second regeneration membranes are shown in Figure 9. In general, it can be seen that the first regenerated membrane is still able to absorb acid numbers, soap levels, and biodiesel levels with a significant value, where the percentage of acid adsorption reaches 66%, soap level reaches 75%, and free glycerol reaches 33.5%. As for the second and third regeneration membrane, the capacity of the IOCM of chitosan–silica to adsorb the impurities present in biodiesel begins to decrease. The adsorption capacity of the IOCM after being regenerated twice could only reduce the acid number by 17% (of the original removal), the soap level by 34%, and free glycerol by 22%. The decrease of the regenerated IOCM in removing biodiesel impurities could be ascribed to the leaching of silica particles due to chitosan swelling [45,46]. As revealed by our investigation on silica particle loading, the impurity adsorptions are dependent on the number of silica particles embedded in the chitosan matrix. However, there is also the possibility of a less effective regeneration performed in this study being responsible for the lower adsorption capacity. 

### 3.6. Biodiesel Filtration 

**Figure 10:**
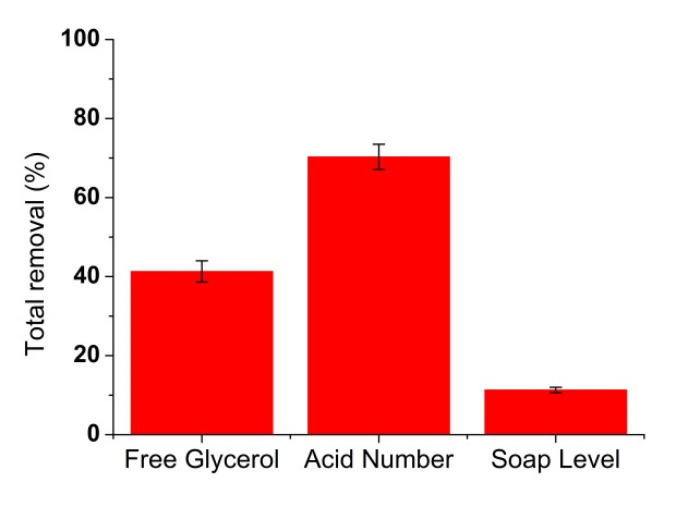
The total removal contaminants in biodiesel by the IOCM of chitosan–silica in the dead-end filtration process.

The IOCM flat sheet has demonstrated its ability as an adsorbent to absorb contaminants in biodiesel and can be used repeatedly. The ability of the IOCM to remove contaminants in biodiesel can also be proven in the biodiesel filtration process. The results of biodiesel filtration using an IOCM using the dead filtration module are shown in Figure 10. Figure 10 shows the total removal of free glycerol, acid number, and soap level contained in biodiesel. The IOCM as an adsorption membrane has the ability to adsorb contaminants contained in biodiesel in the filtration process. Free glycerol can be removed above 41%, and the acid number can be reduced by 70%; therefore, soap content can be removed above 11%. The flux values for clean water and oil filtration were 336 and 275 L/(h m^2^), respectively. The free glycerol content and acid number follow the quality standard of biodiesel, while the decrease in soap content contributes to the improvement of biodiesel quality. The ability of the IOCM to reduce various contaminants in biodiesel shows that IOCMs can be an alternative in the application of conventional dry washing methods for biodiesel purification. IOCMs allow combining filtration and adsorption in one step, which is one of the advantages over conventional dry washing methods. The preparation method of IOCMs is easy and almost any adsorbent can be incorporated into the polymer structure, providing flexibility in improving membrane performance.

## 4. Conclusions

Silica particles extracted from rice husk ash were obtained with properties supporting high impurities uptake from crude biodiesel. The silica particles were then embedded onto the chitosan matrix, resulting in an IOCM with a porous structure. The investigated chitosan–silica IOCM was used in biodiesel purification, with a final product that is acceptable in SNI quality. Contact time and silica particle loading significantly affect the batch purification performance. At optimum contact time and filler loading, the removal successfully reduced the soap level, free glycerol level, and acid number from 547.9 to 12.2 mg/L, 54 to 0.041%, and 2.02 to 1.12 mgKOH/g, respectively. The glycerol removal could be best predicted using the Sips isotherm model. Meanwhile, regeneration remains a challenging factor in this study, though the IOCM demonstrated a good performance in the biodiesel filtration processes. We recommend improving the regeneration method by using the pressure of the methanol to pass through the developed composite membrane. Improving the regeneration of the membrane could help increase the efficiency of biodiesel purification, hence decreasing the overall production cost of biodiesel. 

## Figures and Tables

**Table 1:** Determined isothermal parameters obtained from various models.

Isotherm Model	Equation	Parameters	
Langmuir	$Qe=\frac{Q_{m}K_{L}Ce}{1+K_{l}Ce}$	$R^{2}$	0.996
		RMSE	1.057
		$Q_{m}$	7.749
		$K_{L}$	0.005
Freundlich	$Qe=K_{F}Ce^{1/n}$	$R^{2}$	0.997
		RMSE	0.853
		1/n	1.054
		$K_{F}$	0.045
BET	Qe=QsCBETCe(Cs−Ce)[1+(CBET−1)(CeCs)]	$R^{2}$	0.995
		RMSE	1.113
		$Q_{s}$	2.288
		$C_{BET}$	0.035
		$C_{s}$	2.186
Sips	$Qe=\frac{K_{s}C_{e}^{\beta_{S}}}{1+a_{s}C_{e}^{\beta_{S}}}$	$R^{2}$	0.999
		RMSE	1.77 × 10^−8^
		$K_{s}$	0.037
		$\beta_{S}$	1.00048
		$a_{s}$	0.871

$Q_{m}$ = Langmuir maximum adsorption (mg/g); $K_{L}$ = Langmuir isotherm constant (dm^3^/mg); n = adsorption intensity; $K_{F}$ = Freundlich isotherm constant (mg/g) (dm^3^/g)^n^ related to adsorption capacity; $Q_{s}$ = theoretical isotherm saturation capacity (mg/g); $C_{BET}$ = BET adsorption isotherm relating to the energy of surface interaction (L/mg); $C_{s}$ = adsorbate monolayer saturation concentration (mg/L); $K_{s}$ = Sips isotherm model constant (L/g); $\beta_{S}$ = Sips isotherm model exponent; $a_{s}$ = Sips isotherm model constant (L/mg).

## Data Availability

All data acquired in this study have been presented in this paper.
